# Development of a dissolution method for lumefantrine and artemether in immediate release fixed dose artemether/lumefantrine tablets

**DOI:** 10.1186/s12936-020-03209-5

**Published:** 2020-04-07

**Authors:** Sileshi Belew, Sultan Suleman, Markos Duguma, Henok Teshome, Evelien Wynendaele, Luc Duchateau, Bart De Spiegeleer

**Affiliations:** 1 Jimma University Laboratory of Drug Quality (JuLaDQ) and School of Pharmacy, Jimma University, PO Box 378, Jimma, Ethiopia; 2 Drug Quality and Registration (DruQuaR) Group, Faculty of Pharmaceutical Sciences, Ghent University, Ottergemsesteenweg 460, 9000 Ghent, Belgium; 3 Biometrics Research Group, Faculty of Veterinary Medicine, Ghent University, Salisburylaan 133, B-9820 Merelbeke, Belgium

**Keywords:** Artemether, Dissolution, In-vitro, Lumefantrine

## Abstract

**Background:**

Dissolution of artemether (ART) and lumefantrine (LUM) active pharmaceutical ingredients (APIs) in fixed dose combination (FDC) ART/LUM tablets is one of the critical quality attributes. Thus, the verification of the release profile of ART and LUM from FDC ART/LUM tablets using a robust and discriminatory dissolution method is crucial. Therefore, the aim of this study was to develop and validate an appropriate dissolution method for quality control of FDC ART/LUM tablets.

**Methods:**

The dissolution medium was selected based on saturation solubility data and sink conditions. The effect of agitation speed, pH and surfactant concentration on the release of ART and LUM was evaluated by employing a two-level factorial experiment. The resulting final method was validated for linearity, precision, robustness and API stability. In addition, the discriminatory power of the method was evaluated using expired and unexpired FDC ART/LUM products.

**Results:**

A suitable dissolution profile of FDC ART/LUM tablets was obtained in 900 ml HCl (0.025 N, pH 1.6) with 1%Myrj 52 using paddle method at 100 rpm and 37 °C. ART and LUM were analysed using a HPLC method with UV detection at wavelengths of 210 and 335 nm, respectively. The results from the stability study showed that ART and LUM were sufficiently stable in HCl (0.025 N, pH 1.6) with 1%Myrj 52 at 37 °C. The method was linear (r^2^ = 0.999) over the concentration range of 6.25–100 μg/ml. The results for precision were within the acceptance limit (%RSD < 2). The percent relative standard deviation (< 2%) and statistically non-significant (p > 0.05) difference in release of ART and LUM observed between deliberately changed dissolution method settings (pH = 1.6 ± 0.2 or agitation speed = 100 ± 2) and optimized dissolution conditions revealed the robustness of the dissolution method. The method was capable to discriminate among different FDC ART/LUM products with different quality.

**Conclusions:**

The developed dissolution method is robust and discriminatory. It can be used in the quality evaluation of FDC ART/LUM tablets.

## Background

Artemether (ART) and lumefantrine (LUM) are anti-malarial agents that demonstrate synergistic anti-malarial activity resulting in rapid clearance of parasitaemia and prevention of recrudescence [1–5]. Thus, fixed dose combination (FDC) ART/LUM (20 mg/120 mg) products are widely used as the first-line treatment for uncomplicated *Plasmodium falciparum* malaria [6–9]. ART (logP 3.53) and LUM (logP 9.19, pKa 8.73 and 13.49) [10] are classified as Biopharmaceutical Classification System (BCS) class IV drugs [11]. Hence, permeability and solubility/dissolution are critical attributes [12, 13] that can influence the rate and extent of drug absorption and bioavailability [14–16].

FDC ART/LUM products are available as solid oral dosage forms like tablets. The dissolution of drugs can be influenced by various API and formulation factors, such as particle size variability of the APIs or granulation technology [17–21], as well as by dissolution-method variables, such as pH, buffer capacity, ionic strength and presence of surfactants [22]. Hence, it is important to establish the in vitro dissolution conditions that help to discriminate significant ingredients and manufacturing process variability in an attempt to assure quality consistency, and possibly guarantee also to some extent the in vivo performance, of FDC ART/LUM solid oral dosage forms.

Dissolution methods for FDC ART/LUM tablets are currently missing in official monographs and available methods described in the literature need relative long dissolution times (120 min), use independent dissolution conditions for each API or inadequately consider the relative effect of dissolution method variables [23, 24]. Consequently, developing an appropriate dissolution method for FDC ART/LUM products is crucial. The present study was therefore aimed to develop and validate a QC-relevant, efficient, robust and discriminatory dissolution method for ART and LUM in FDC ART/LUM tablets.

## Methods

### Drugs/chemicals/reagents/solvents

ART and LUM working reference standards were obtained from Dafra Pharma International (B-2300 Turnhout, Belgium) through Drug Quality and Registration (DruQuaR) laboratory, University of Ghent, Belgium. Ultrapure water (18.2 MΩ. cm at 25 °C) was prepared in Jimma University Laboratory of Drug Quality (JuLaDQ) using Nanopure Analytical ultrapure water system (model number: D11901 (7143), Thermo fisher Scientific). Acetonitrile (HPLC grade, Fisher Scientific), Tween 80 (AcrosOrganics), Benzalkonium chloride (Fagron), Sodium lauryl sulfate (Sigma-aldrich), Polyoxyl 40 Stearate (Myrj 52) (Sigma-aldrich), Hydrochloric acid (Sigma-aldrich), Orthophosphoric acid (Fluka) and Tetrahydrofuran (HPLC grade, Sigma-aldrich) were used as received. FDC ART/LUM (20 mg ART/120 mg LUM) products were used. Detailed information on the five FDC ART/LUM products investigated in this study is presented in Additional file 1: Table S1.

## Experimental

### Mass uniformity

The mass uniformity of tablet samples of each brand of FDC ART/LUM products was conducted according to the method given in the European Pharmacopoeia [25]. Randomly selected tablets (n = 20) were individually weighed with a calibrated balance (Mettler Toledo, AL204-1C, Switzerland). The results were evaluated against the European Pharmacopoeia specifications (i.e. the deviation of individual masses from average mass should not exceed ± 7.5%, with only maximum 2 tablets allowed to deviate maximally ± 15%).

### Amount of active compound

The amount of ART and LUM in samples of FDC ART/LUM tablets was determined based on the previously published HPLC method [26]. In brief, the analysis of ART and LUM was conducted using Agilent 1260 Infinity Series HPLC system (Agilent Technologies, Santa Clara, California, USA) equipped with a Halo-RP-Amide column (50 × 4.6 mm, 2.7 µm) coupled to a diode-array detector (DAD). The detection wavelengths of ART and LUM were 210 and 335 nm, respectively. The flow rate, injection volume, run time and column temperature were 1 ml/min, 3 µl, 5 min and 30 °C, respectively. The mobile phase used was a mixture of acetonitrile/0.001 M potassium phosphate buffer pH 3.0 (52:48% v/v).

### System suitability tests (SST)

System suitability for analysis of ART and LUM was evaluated according to the European Pharmacopoeia method [27]. The symmetry factor (A_s_) of principal peaks was calculated using the following formula:

$$As = Wx/2d$$

where W_x_ = peak width at 5% of reference standard peak height measured from the base line, d = base line distance between the perpendicular dropped from the peak maximum and the leading edge of the peak at 5% of peak height measured in the same unit as W_x_. The specification was an A_s_ value of maximally 1.5. In addition, percent relative standard deviation (%RSD) of replicate injections (n = 6) of reference standards were calculated and compared against the European Pharmacopoeia specification limit (i.e. %RSD of six injections should be ≤ 1.2).

### Preparation of ART and LUM standard solutions

Working reference standard of ART (20 mg) and LUM (120 mg) were individually added into a 100.0 ml volumetric flask, dissolved in 80 ml tetrahydrofuran (HPLC grade, Sigma-aldrich), sonicated for 15 min, filled to volume with mobile phase (acetonitrile/0.001 M potassium phosphate buffer pH 3.0 (52:48% v/v)), filtered using 0.45 µm Whatman filter paper (CAT No 1102090) and analysed using HPLC.

### Preparation of sample solutions

Tablet samples (n = 20) were weighed and grounded into fine powder with clean and dry mortar and pestle [28]. An accurately weighed portion of powder equivalent to 20 mg ART and 120 mg LUM was individually added into 100.0 ml volumetric flask, dissolved in 80 ml of tetrahydrofuran (HPLC grade, Sigma-aldrich), sonicated for 15 min, filled to volume with mobile phase (acetonitrile/0.001 M potassium phosphate buffer pH 3.0 (52:48% v/v)), filtered using 0.45 µm Whatman filter paper (CAT No 1102090) and analysed using HPLC.

### Equilibrium solubility

An equilibrium solubility study was conducted by the shake-flask method [29]. LUM (10 mg) was added into 50 ml conical flask and dissolved in 10 ml 1% w/v surfactant/HCl (pH 1.2) and 1–2% w/v surfactant/HCl (pH 2.3), while ART (10 mg) was added into 50 ml conical flask and dissolved in 0.5–2% surfactant/HCl (pH 1.2 or 2.3) and 0.5–1% w/v surfactant/buffer (pH 4.5 or 6.8). The flasks were incubated at 37 °C for 24 h whilst shaking at 100 rpm. The samples were rapidly filtered using 0.45 µm PVDF (polyvinylidene fluoride) syringe filter, suitably diluted (2.5 ± 0.5 times) with acetonitrile and analysed at the auto-sampler temperature of 37 *°*C using HPLC system (Waters Alliance 2695 Separations Module Milford, MA, USA) equipped with a Halo-RP-Amide column (50 × 4.6 mm, 2.7 µm) as described in previous HPLC method [26].

### Solution stability

The solution stability was evaluated by analysing the concentration of ART and LUM (mixture) in dissolution medium stored at 37 *°*C for at least 24 h.

### Dissolution of ART and LUM from FDC ART/LUM tablets

#### Screening study

Screening experiments were conducted using dissolution medium (Myrj 52/HCl) selected based on the results of equilibrium solubility of ART and LUM. Agitation speed (A1), pH (A2) and surfactant concentration (A3) were the factors used for the release of ART and LUM. Three-way factorial (each factor with two levels) experiment (Design Expert 6.0.1 software (Stat Ease. Inc.)) was employed to determine the best combination of factors for the release of ART and LUM. The factor settings for Design of Experiment (DoE) are presented in Additional file 2: Table S2. The dissolution was conducted using USP type II (Paddle) method (RC-8 dissolution apparatus, China). The volume of dissolution medium and bath temperature were 900 ml and 37 ^°^C, respectively. FDC ART/LUM tablets (Ipca Laboratories, India, batch no. DYI 478058, within shelf-life period) (n = 2) were subjected to the different dissolution conditions. Samples (10 ml) were withdrawn at 30, 60, 90 and 120 min, filtered through 0.45 µm Whatman filter paper (CAT No 1102090), diluted with acetonitrile and analysed using the HPLC method [26]. Agilent 1260 Infinity series HPLC system coupled with a Halo-RP-Amide column (50 × 4.6 mm, 2.7 µm) and diode-array detector (DAD) was used. The mobile phase used was acetonitrile (HPLC grade, Sigma-Aldrich)/(0.001 M potassium phosphate buffer pH 3.0) (58:42% v/v). The detection wavelengths used for the analysis of ART and LUM were 210 and 335 nm, respectively. The sample temperature in the auto-injector was 37 °C. The column temperature, flow rate, injection volume, run time were 30 °C, 1.5 ml/min, 20 µl and 12 min, respectively.

#### Optimization of dissolution conditions

Since the target dissolution of both ART and LUM from FDC ART/LUM tablets was set at Q ≥ 80% at 60 min, optimal dissolution conditions giving the desired response were selected and optimized using a desirability approach [Design Expert 6.0.1 software (Stat Ease. Inc.)].

#### Discriminatory power

The discriminatory power of the optimized dissolution conditions was evaluated using commercially available FDC ART/LUM products (4 unexpired, before the labelled expiry date, and 1 expired, beyond the labelled expiry date). Area under the dissolution curve (AUC), dissolution efficiency (DE) and mean dissolution time (MDT) of different FDC ART/LUM products were estimated using KinetDS software program (KinetDS 3.0). The results were compared using ANOVA based data evaluation. In addition, release (%) of ART and LUM at 60 min from different FDC ART/LUM products was compared using post hoc multiple comparison test.

#### Validation of the dissolution procedures

The applied HPLC method was based on a previously developed and validated method for the assay of both API in tablets [26]. As in this study, dissolution samples were to be analysed, as well as the operational conditions were slightly adapted to meet the SST, the adapted method was revalidated.

##### Linearity and range

The HPLC method [26] used for the quantification of ART and LUM in the dissolution samples was evaluated for linearity by analyzing the concentrations of ART and LUM ranging from 6.25 to 100 μg/ml. The regression line was assessed by determining the 95% confidence interval (95% CI) of slope and intercept parameters as well as by evaluating F-lack of fit and the residual plot.

##### Precision/repeatability

Precision was determined by repeatability and intermediate precision studies. Repeatability of the method was done by multiple measurements (n = 6) of the sample of tablets by the same analyst, while intermediate precision was done by performing the dissolution test on the same sample of tablets on different days by at least two analysts. The results were compared against the acceptance limits given in the European Pharmacopoeia for assaying APIs (%RSD ≤ 1.2) [27].

##### Robustness

Robustness was studied by evaluating the effect of small but deliberate variations (i.e. the pH of the dissolution medium (± 0.2) and paddle rotation speed (± 2 rpm)) in the optimized dissolution conditions. Percent relative standard deviation (%RSD) was calculated and compared against the suggested  %RSD (< 2) [30]. Comparative statistical analysis of the results obtained from two dissolution conditions (i.e. optimized vs. optimized with deliberate change in parameters) was performed using Student’s *t* test (p < 0.05).

### Statistical analysis

Statistical analysis was performed using Statistix-8 software and SPSS version 20.

## Results

### Mass uniformity

The results of mass uniformity (mean: 244.00 to 291.95 and SD: 1.52 to 9.67, n = 20) of five commercially available FDC ART/LUM products revealed that all products comply with the European Pharmacopoeia specification limits (i.e. the deviation of individual masses from average mass should not exceed ± 7.5%, with only maximum 2 tablets allowed to deviate maximally ± 15%). The results of mass uniformity of five commercially available FDC ART/LUM products are presented in Additional file 3: Table S3.

### Amount of active compounds

The results of amount of ART and LUM in FDC ART/LUM tablets is presented in Table 1. The assay results revealed that all samples comply with the generally accepted specification criteria for both ART and LUM, i.e. percentage label claim (%l.c.) between 90 and 110%.

**Table 1:** Results of amount of active compounds in FDC ART/LUM tablets

Product	ART	LUM
	% l.c. (%RSD)	% l.c. (%RSD)
Comether^®^	104.6 (0.73)	109.3 (0.72)
Artel-L^®^	108.1 (1.06)	103.2 (2.15)
Artemine^®^	104.7 (2.92)	106.1 (0.04)
ART/LUM	110.1 (0.34)	109.8 (0.25)
ART/LUM-E^a^	90.1 (0.66)	95.8 (0.63)

^a^Expired product, l.c.: label claim

### Equilibrium solubility

The results of the equilibrium solubility of ART and LUM are presented in Table 2. The results revealed that relatively higher solubility of ART and LUM was observed in 1–2% Myrj 52/acidic pH. This reflects that the presence of surfactant in acidic pH enhances solubility of both APIs. Both APIs were stable in the dissolution medium for 24 h. The percentage differences obtained (ART: 1.5–1.8%, LUM: 0.9–1.4%) at the end of 24 h from the initial (0 h) value suggest stability of both compounds in the dissolution medium.

**Table 2:** Results of ART and LUM APIs solubility in different media as determined by HPLC

#	Medium	Solubility (µg/ml)
*ART*		
1	0.5% w/v Myrj 52/HCl (pH 1.2)	151
2	1% w/v Myrj 52/HCl (pH 1.2)	181
3	1% w/v Myrj 52/HCl (pH 2.3)	290
4	1.5% w/v Myrj 52/HCl (pH 2.3)	452
5	2% w/v Myrj 52/HCl (pH 2.3)	652
6	0.5% w/v SLS/HCl (pH 1.2)	323
7	1% w/v SLS/HCl (pH 1.2)	337
8	0.5% w/v Myrj 52/0.05 M ammonium acetate buffer (pH 4.5)	8
9	1% w/v Myrj 52/0.05 M ammonium acetate buffer (pH 4.5)	33
10	0.5% w/v SLS/0.05 M ammonium acetate buffer (pH 4.5)	138
11	1% w/v SLS/0.05 M ammonium acetate buffer (pH 4.5)	219
12	0.5% w/v Myrj 52/0.05 M sodium phosphate buffer (pH 6.8)	22
13	1% w/v Myrj 52/0.05 M sodium phosphate buffer (pH 6.8)	37
14	0.5% w/v SLS/0.05 sodium phosphate buffer (pH 6.8)	5
15	1% w/v SLS/0.05 M sodium phosphate buffer (pH 6.8)	5
16	0.5% w/v Tween80/0 0.05 M ammonium acetate buffer (pH 4.5)	24
17	1% w/v Tween 80/0.05 M ammonium acetate buffer (pH 4.5)	23
18	0.5% w/v Tween 80/HCl (pH 1.2)	10
19	1% w/v Tween 80/HCl (pH 1.2)	17
*LUM*		
20	1% w/v Myrj 52/HCl (pH 2.3)	626
21	1.5% w/v Myrj 52/HCl (pH 2.3)	813
22	2% w/v Myrj 52/HCl (pH 2.3)	1033
23	1% w/v Tween 80/HCl (pH 1.2)	112
24	1% w/v Benzalkonium chloride/HCl (pH 1.2)	93
25	1% w/v Sodium lauryl sulfate/HCl (pH 1.2)	59

### Dissolution of ART and LUM from FDC ART/LUM tablets

#### Screening study

**Fig. 1:**
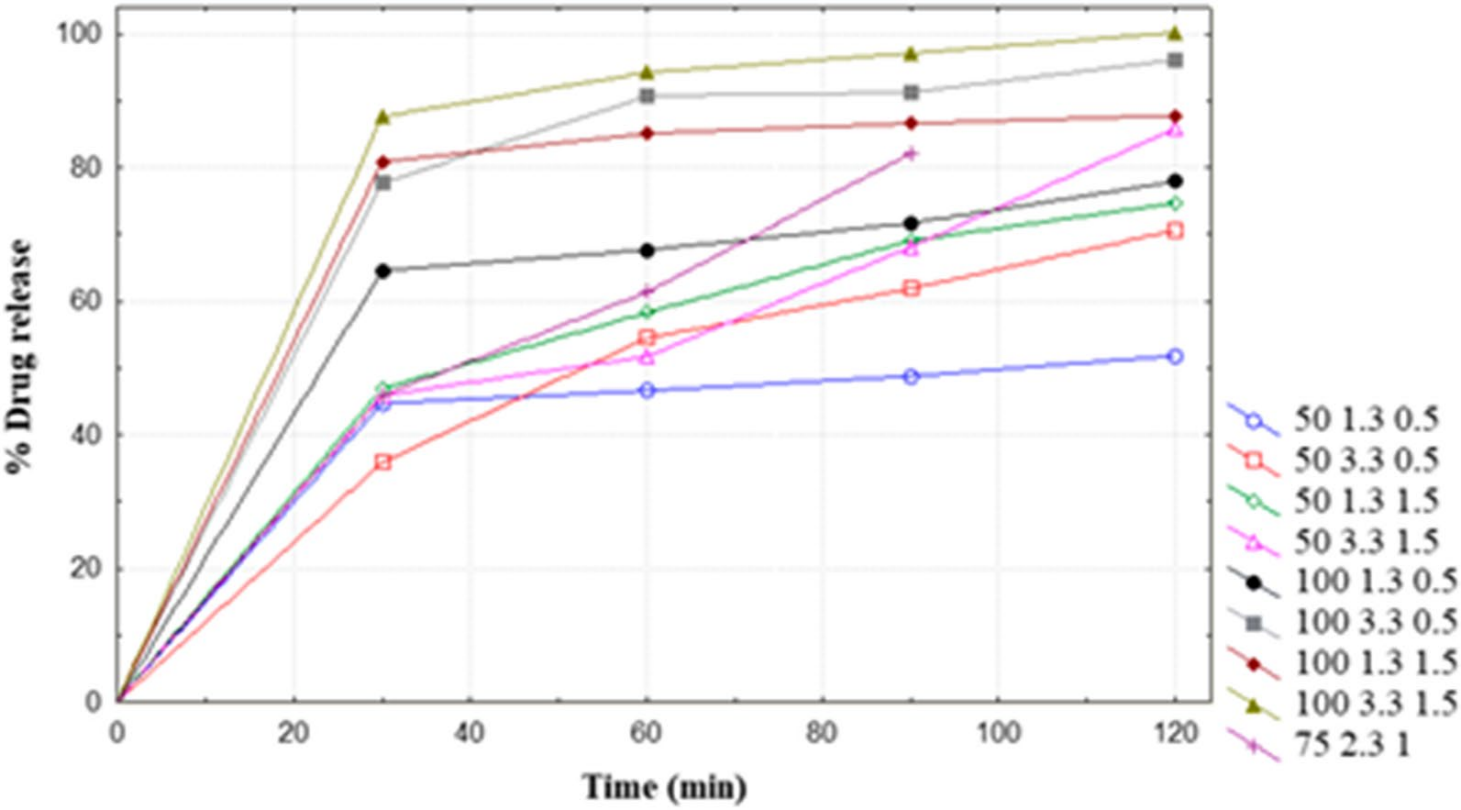
Dissolution profile (mean, n = 2) of ART API in FDC ART/LUM tablets (Ipca Laboratories, India, batch no. DYI 478058) in 900 ml dissolution media at 37 ± 0.5 °C) using USP apparatus II (paddle). (Legend from left to right: rpm pH Myrj52 concentration)

**Fig. 2:**
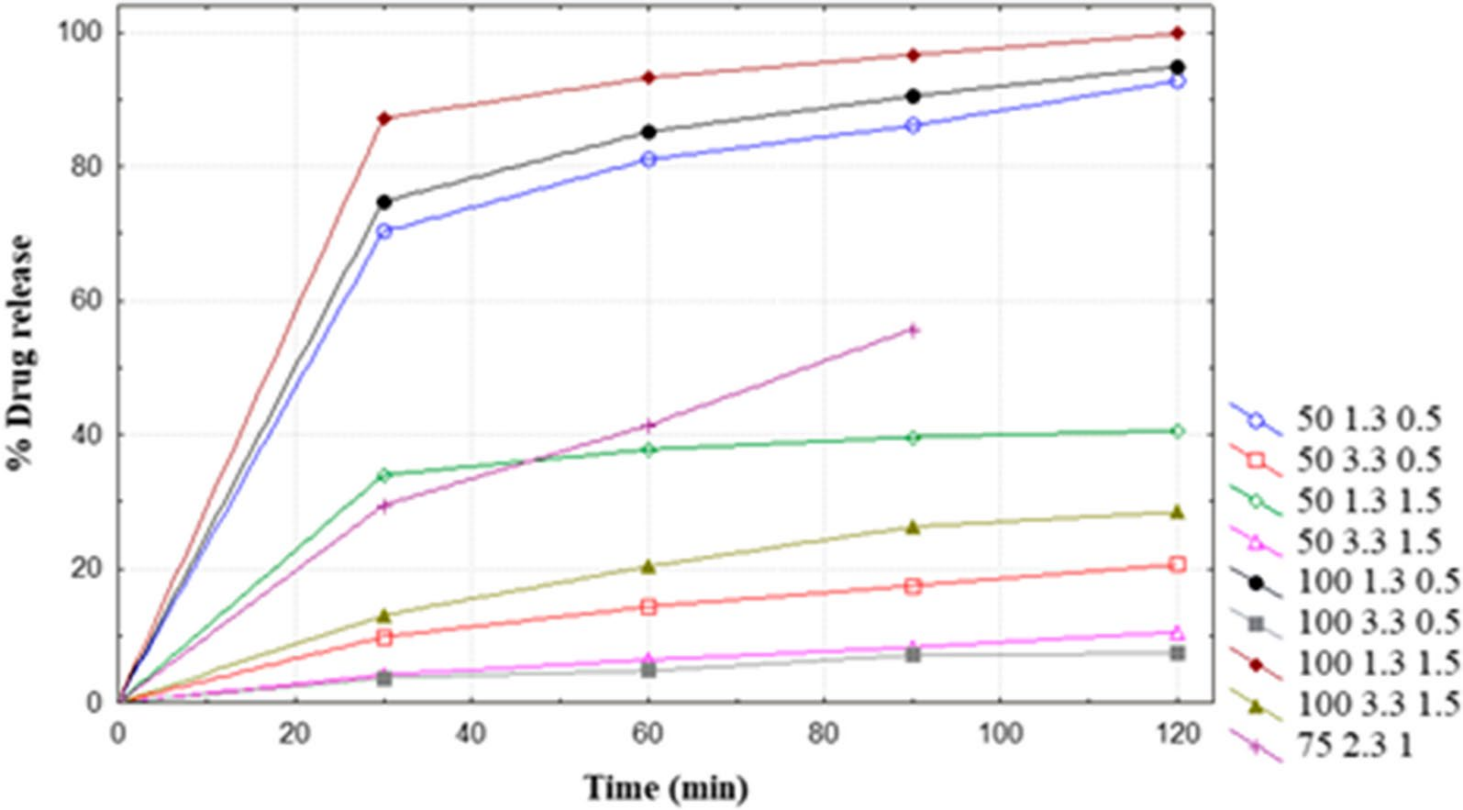
Dissolution profile (mean, n = 2) of LUM API in FDC ART/LUM tablets (Ipca Laboratories, India, batch no. DYI 478058) in 900 ml dissolution media at 37 ± 0.5 °C using USP apparatus II (paddle). (Legend from left to right: rpm pH Myrj52 concentration)

The individual response results of the DoE experiment are presented in Additional file 4: Table S4. Typical dissolution profiles of ART and LUM from unexpired FDC ART/LUM tablets under the different DoE-experimental conditions are presented in Figs. 1 and 2, respectively. At 60 min, maintaining the agitation speed at 100 rpm and surfactant concentration at 1.5% in the DoE-experimental conditions, decreasing the pH of the dissolution medium by 2 units (3.3 to 1.3) increased the dissolution of LUM from 13.0% to 87.2%, and slightly decreased the dissolution of ART from 87.6 to 80.9%.

The equations for response factor (% release at 60 min) of ART and LUM are given below.

1 $$Y1 = 68.6 + 15.8A1 + 4.2A2 + 3.7A3 + 3.8A1A2 + 1.5A1A3 - 3.8A2A3$$

2 $$Y2 = 42.8 + 8.2A1 - 31.4A2 - 3.3A3 - 7.1A1A2 + 9.3A1A3 + 5.2A2A3 - 3.4A1A2A3$$

Where Y1 and Y2 are % release of ART and LUM, respectively. A1, A2 and A3 are coded variables with two levels representing agitation speed (50 to100 rpm, corresponding to coded A1 values − 1 to + 1), pH (1.3 to 3.3 corresponding to coded A2 values − 1 to + 1,) and surfactant concentration (0.5 to 1.5%, corresponding to coded A3 values − 1 to + 1), respectively.

The model used to fit the response variable (i.e. release of ART and LUM at 60 min) was significant (p < 0.0001) to represent the relationship between the response and the independent variables. The model *F*-value (ART: 119, LUM: 2107) suggested significance of the model. The R^2^ value (ART: 0.996, LUM: 0.999) indicated that only 0.4 (ART) and 0.1% (LUM), of the total variation of response data was not explained by the model. Agitation speed (A1), (Eq. 1), respectively pH (A2) (Eq. 2), demonstrated a relatively higher effect on the release of ART, respectively LUM, than the other variables.

The results of the effects of independent factors on the release of ART and LUM from FDC ART/LUM product (Ipca Laboratories, India, batch no. DYI 478058) are presented in Tables 3 and 4, respectively.

**Table 3:** Effect of independent factors on the release of ART from FDC ART/LUM tablets

Time (min)	Factor settings			Mean (%) release
	A1	A2	A3	
30	100	3.3	1.5	87.65^a^
	100	1.3	1.5	80.88^ab^
	100	3.3	0.5	77.70^b^
	100	1.3	0.5	64.58^c^
	50	1.3	1.5	46.92^d^
	50	3.3	1.5	45.87^d^
	50	1.3	0.5	44.72^de^
	50	3.3	0.5	35.86^e^
	LSD (0.05)			9.3
	CV			3.88
	p-value			0.01

	A2	A3	Mean (%) release
60	3.3	1.5	73.00^a^
	3.3	0.5	72.63^a^
	1.3	1.5	71.71^a^
	1.3	0.5	57.15^b^
	LSD (0.05)		6.36
	CV		4.1
	p-value		0.001
	A1	A2	Mean (%) release
	100	3.3	92.52^a^
	100	1.3	76.41^b^
	50	3.3	53.12^c^
	50	1.3	52.45^c^
	LSD (0.05)		6.36
	CV		4.1
	p-value		0.001

	A2	A3	Mean (%) release
90	3.3	1.5	82.59^a^
	1.3	1.5	77.83^b^
	3.3	0.5	76.56^b^
	1.3	0.5	60.73^c^
	LSD (0.05)		4.14
	CV		2.46
	p-value		0.000
	A1	A2	Mean (%) release
	100	3.3	94.17^a^
	100	1.3	79.15^b^
	50	3.3	64.98^c^
	50	1.3	59.42^d^
	LSD (0.05)		6.36
	CV		4.1
	p-value		0.000

	A2	A3	Mean (%) release
120	3.3	1.5	93.03^a^
	3.3	0.5	83.40^b^
	1.3	1.5	81.26^b^
	1.3	0.5	65.69^c^
	LSD (0.05)		4.47
	CV		2.44
	p-value		0.01
	A1	A3	Mean (%) release
	100	1.5	94.01^a^
	100	0.5	87.05^b^
	50	1.5	80.27^c^
	50	0.5	62.03^d^
	LSD (0.05)		4.47
	CV		2.44
	p-value		0.000

Means with the same letters are not significantly different from each other

**Table 4:** Effect of independent factors on the release of LUM from FDC ART/LUM tablets

Time (min)	Factor settings			Mean (%) drug release
	A1	A2	A3	
30	100	1.3	1.5	87.24^a^
	100	1.3	0.5	74.81^b^
	50	1.3	0.5	70.29^b^
	50	1.3	1.5	33.98^c^
	100	3.3	1.5	13.05^d^
	50	3.3	0.5	9.82d^e^
	50	3.3	1.5	4.14^e^
	100	3.3	0.5	3.68^e^
	LSD (0.05)			8.84
	CV			6.02
	p-value			0
60	100	1.3	1.5	93.27^a^
	100	1.3	0.5	85.2^b^
	50	1.3	0.5	81.08^b^
	50	1.3	1.5	37.77^c^
	100	3.3	1.5	20.36^d^
	50	3.3	0.5	14.32^e^
	50	3.3	1.5	6.39^f^
	100	3.3	0.5	4.76^f^
	LSD (0.05)			5.37
	CV			3.17
	p-value			0
90	100	1.3	1.5	96.68^a^
	100	1.3	0.5	90.52^b^
	50	1.3	0.5	86.11^b^
	50	1.3	1.5	39.63^c^
	100	3.3	1.5	26.23^d^
	50	3.3	0.5	17.44^e^
	50	3.3	1.5	8.25^f^
	100	3.3	0.5	7.07^f^
	LSD (0.05)			5.56
	CV			3.03
	p-value			0
120	100	1.3	1.5	99.83^a^
	100	1.3	0.5	94.96^ab^
	50	1.3	0.5	92.83^b^
	50	1.3	1.5	40.58^c^
	100	3.3	1.5	28.47^d^
	50	3.3	0.5	20.61^e^
	50	3.3	1.5	10.56^f^
	100	3.3	0.5	7.39^f^
	LSD (0.05)			5.14
	CV			2.63
	p-value			0

Means with the same letters are not significantly different from each other

Considering the results at 60 min, at lower pH (1.3), the change in surfactant concentration from 1.5 to 0.5% decreased release of ART (71.7 to 57.1%). In addition, at high agitation speed (100 rpm), the change in pH from 3.3 to 1.3 decreased release of ART (92.5 to 76.4%). For LUM, at high agitation speed (100 rpm) and low pH (1.3), change in surfactant concentration from 1.5 to 0.5% decreased its release from 93.3 to 85.2%.

**Fig. 3:**
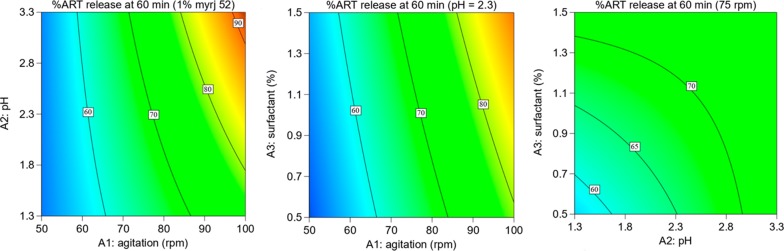
Contourplots showing the influence of interaction of factorson release (%) of ART API at 60 min

**Fig. 4:**
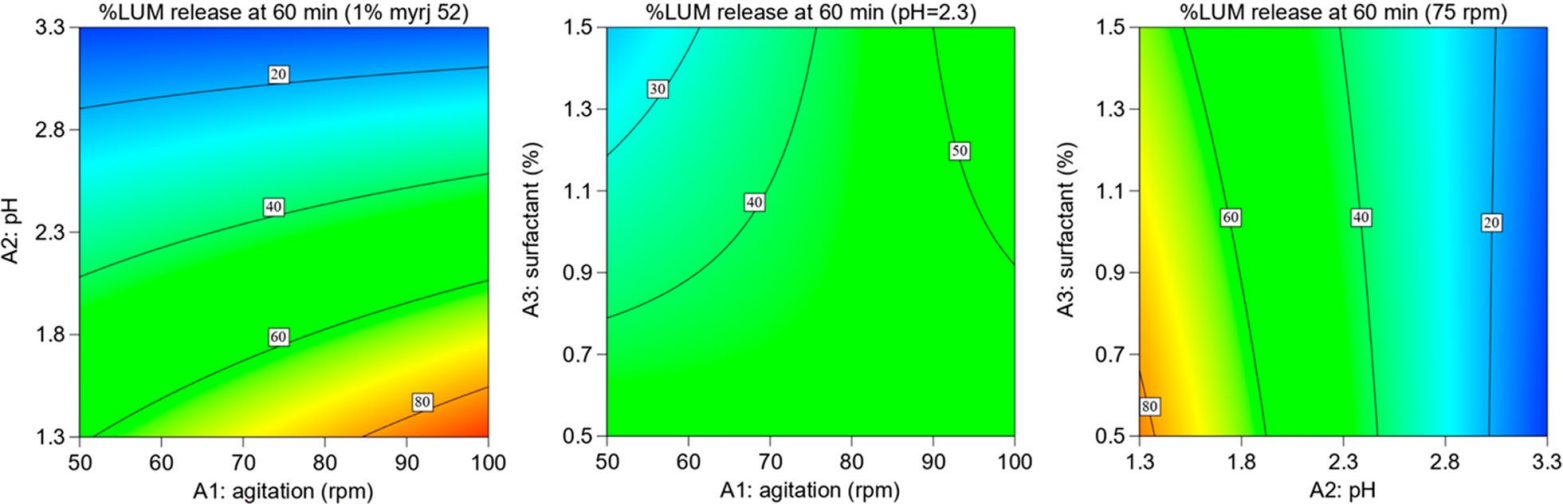
Contour plots showing the influence of interaction of factors on release (%) of LUM API at 60 min

Contour plots showing the interaction effect of factors on release of ART and LUM at 60 min are presented in Figs. 3 and 4, respectively. Figure 3 shows that an increase in agitation speed from 60 to 100 rpm with increase in pH from 2.3 to 3.3 increased the release (%) of ART. Figure 4 shows that an increase in agitation speed from 70 to 100 rpm and a decrease in pH from 1.8 to 1.3 increased the release (%) of LUM.

#### Discriminatory power

**Fig. 5:**
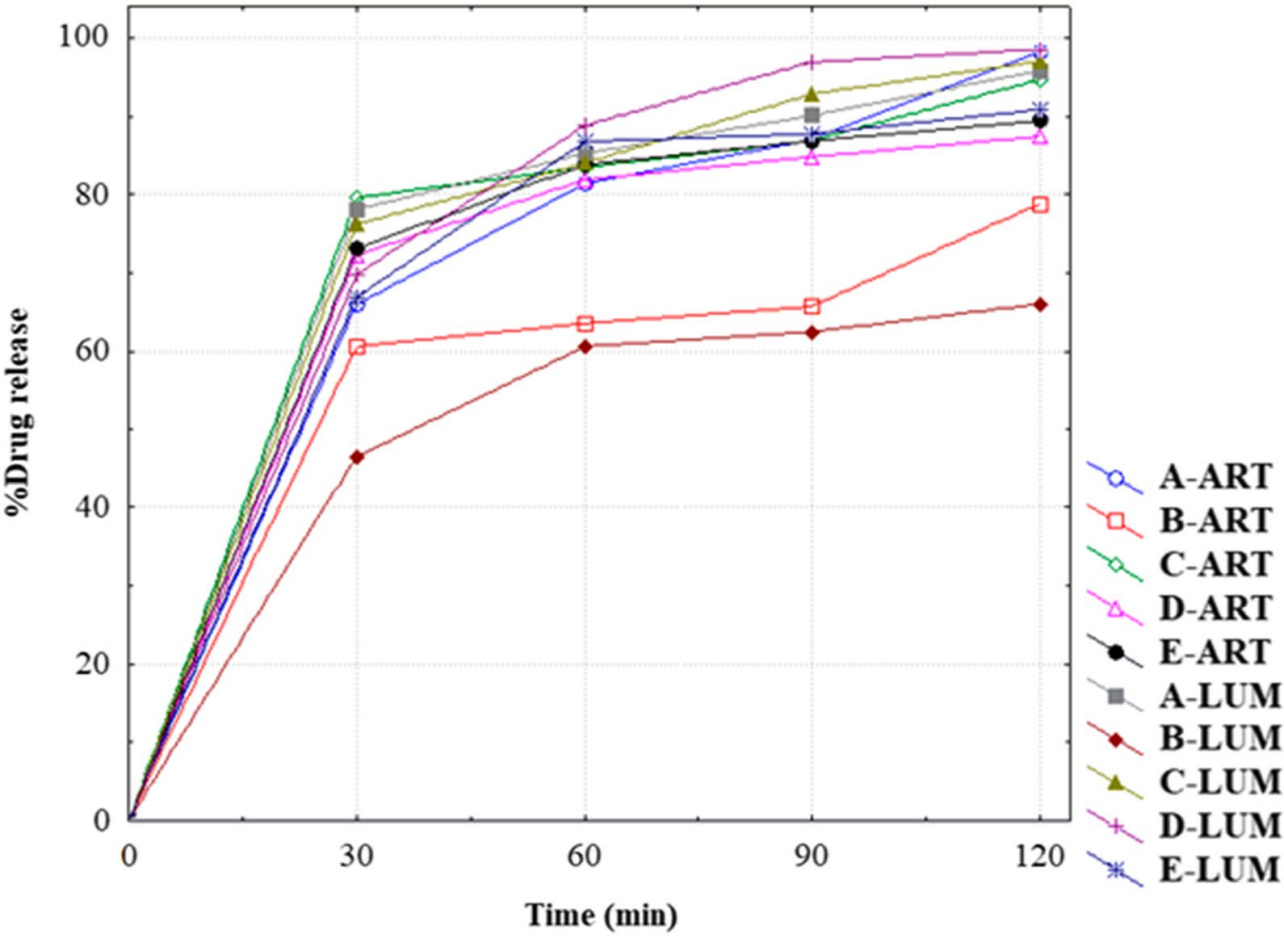
Dissolution profile (n = 6) of ART and LUM APIs in FDC ART/LUM tablets in 900 ml (1% Myrj 52/HCl (pH 1.6) at 37 ± 0.5 °C, 100 rpm) using USP II apparatus. **a** ART/LUM (unexpired), **b** Comether^®^, **c** Artemine^®^, **d** Artel-L^®^ and **e** ART/LUM-E (expired) products

The release profiles of ART and LUM from FDC ART/LUM products, subjected to the same dissolution conditions, are presented in Fig. 5. Post-hoc multiple comparisons test on release (%) (at 60 min) of ART and LUM from different FDC ART/LUM products are presented in Additional file 5: Table S5. The results of 95% CI for the mean (%) release of ART and LUM at different time points are presented in Additional file 6: Table S6. At 60 min, the release of ART from different FDC ART/LUM products ranges from 63.60% (95% CI 62.62–64.58) to 83.83% (95% CI 82.75–84.91), respectively, while the release of LUM ranges from 60.68% (95% CI 59.33–62.03) to 88.82% (95% CI 86.29–91.34%).

The results of pair-wise comparison of means of area under the dissolution curve (AUC), dissolution efficiency (DE) and mean dissolution time (MDT) of ART and LUM from different FDC ART/LUM products estimated using KinetDS software program (KinetDS 3.0) are presented in Additional file 7: Table S7.

#### Validation of the method

Linearity. The linearity calibration curve for the method indicated the fitness-for-use of the applied method. The 95% CI for the regression slope (2.81, 95% CI 2.73 to 2.88), y-intercept (− 4.99, 95% CI − 1.05 to − 8.93) and ANOVA F-value of 13680.79 of ART and the 95% CI for the regression slope (21.35, 95% CI 20.93 to 21.77), y-intercept (− 22.33, 95% CI 20.93 to 44.13) and ANOVA F-value of 25871.1 as well as R^2^ value of 0.999 proved a strong positive linear relationship. In addition, random pattern of the residual plot showed a good fit of the linear model to the data.

##### Precision/repeatability

The %RSD for repeatability of the HPLC method used for the quantification of ART and LUM in dissolution samples at 60 min was within the specification limit (%RSD ≤ 1.2). The results of %RSD for the repeatability and intermediate precision are presented in Table 5.

**Table 5:** Dissolution results at 60 min of ART and LUM from FDC ART/LUM tablets

#	% Drug release (n = 6)			
	Analyst 1	Analyst 2	Analyst 1	Analyst 2
	ART	ART	LUM	LUM
1	85.27	80.24	84.63	81.22
2	82.63	84.41	82.02	82.85
3	82.42	82.44	85.73	88.82
4	83.60	85.42	83.53	87.01
5	82.42	82.44	81.46	82.17
6	86.78	83.12	86.34	85.23
Mean	83.85	83.01	83.95	84.55
SD	1.8	1.79	1.97	2.98
%RSD	2.15	2.16	2.35	3.52
Mean	83.43		84.25	
SD	0.59		0.42	
%RSD	0.35		0.50	

FDC ART/LUM tablets (Ipca Laboratories, India, batch no. DYI 478058) were used

##### Robustness

The %RSD values (< 2%) and the statistically non-significant (p > 0.05) difference between the release (%) profile of ART and LUM APIs in two dissolution conditions (optimized vs. optimized with deliberate change in parameters) revealed the robustness of the dissolution conditions. The results of release profile of ART and LUM are presented in Table 6.

**Table 6:** Dissolution (n = 6) of ART and LUM from FDC ART/LUM tablets

Time (min)	pH						Agitation speed (rpm)					
	ART			LUM			ART			LUM		
	1.4	1.6	1.8	1.4	1.6	1.8	98	100	102	98	100	102
30	73.2	75.7	80.0	77.1	79.6	78.7	77.4	79.3	76.9	73.0	78.1	72.6
60	80.9	84.6	85.9	88.2	85.8	84.8	82.2	85.7	84.7	83.2	84.6	85.9
90	89.0	91.3	92.9	91.1	95.5	96.1	88.8	90.4	87.6	92.9	96.0	96.4
120	94.0	94.7	96.5	95.2	98.1	99.0	89.5	93.3	91.7	98.9	99.1	98.9
Average at 60 min	83.81 ± 2.62			86.24 ± 1.75			84.20 ± 1.79			84.59 ± 1.34		
%RSD	3.13			2.04			0.02			0.01		
p > 0.05												

FDC ART/LUM tablets (Ipca Laboratories, India, batch no. DYI 478058) were used. All dissolution conditions except the deliberately changed parameters were kept constant

## Discussion

Dissolution testing is an important analytical tool used to verify the release of API from solid oral dosage forms and evaluate the impact of formulation composition and process parameters on the in vitro release of API [31, 32]. In the present study, a dissolution method for the in vitro dissolution of ART and LUM simultaneously from FDC ART (20 mg)/LUM (120 mg) tablets is developed.

The results of saturation solubility indicate that the solubility of ART and LUM in acidic pH (2.3) with 1% Myrj 52 was 290 and 626 µg/ml, respectively. This reflects that this medium maintains the sink conditions of both APIs that could ensure the minimum solubility required for ART (60 µg/ml) and LUM (360 µg/ml) from FDC ART (20 mg)/LUM (120 mg) tablets in 900 ml volume dissolution medium. The results of solution stability indicate that both APIs are stable in the selected dissolution medium at 37 °C. Since the factorial design helps to study the independent and interactions effects of factors [33], three factors (i.e. agitation speed, pH and surfactant) were evaluated by employing two level factorial design. From the equation of the model obtained using the experimental results of release of ART and LUM at 60 min, it is seen that, keeping all other terms constant, a unit increase (50 rpm: − 1 to + 1) in agitation speed could increase the release of ART by 15.8%. While a unit increase in pH could decrease the release of LUM by 31.4%. This suggests that agitation speed has a relatively strong influence on the release of ART, while pH is the most important variable for LUM.

The results obtained from the ANOVA-based data evaluation applied to different FDC ART/LUM products revealed differences in release of ART and LUM at 60 min, AUC, DE and MDT among FDC ART/LUM products. This points to differences in pharmaceutical attributes (API, formulation and/or manufacturing processes) among the different FDC ART/LUM products and suggests the discriminatory power of the developed dissolution method. Therefore, the developed dissolution method is considered appropriate for testing batch-to-batch quality consistency [34, 35]. In addition, a discriminatory dissolution method might in principle reflect the in vivo performance of drugs. However, since factors governing in vitro and in vivo drug release are not similar [36–38], the in vitro release profile of a product is not necessarily related to its in vivo behavior. Especially for BCS (biopharmaceutical classification system) class IV drugs, such as ART and LUM, there is often not a well-established correlation. In addition, the typical media volume (900 ml) has little bio-relevance as fasted gastric and intestinal volume is about 500 ml, and differences in hydrodynamic mixing efficiency were observed using different volumes [39].

The %RSD values (< 2) and the statistically non-significant (p > 0.05) difference between release of ART or LUM in two dissolution conditions (optimized vs. modified with deliberate changes in parameters), in the present study reflect that the developed method is robust enough to allow normal variability in routine testing. This implies that at normal operation conditions, the developed method could not lead to unnecessary rejection of products. The proposed dissolution method allowing the simultaneous dissolution profiling of ART and LUM in FDC ART/LUM tablets is using the USP apparatus II (paddle) in HCl (0.025 N, pH 1.6) with 1% Myrj 52 as dissolution medium at 100 rpm and 37 °C. Based on the results of release of ART and LUM in commercially available FDC ART/LUM tablets subjected to the developed dissolution method, Q ≥ 80% at 60 min is suggested as a quality acceptance limit for the dissolution test of ART and LUM from FDC ART/LUM tablets, which is consistent as the QC quality attribute of most immediate-release products [40]. Since dissolution test of ART and LUM is currently missing in an official monographs, the developed dissolution method could be considered suitable for quality control and dissolution profile comparison of different commercial formulations of FDC ART/LUM products.

## Conclusion

The results of the present study revealed that the developed and validated dissolution method with HPLC–UV determination was capable to distinguish significant formulation variations and allow normal variability in routine testing. Therefore, it is suitable for simultaneous dissolution testing of ART and LUM from FDC ART/LUM tablets and can be effectively applied in quality control.

## Supplementary information

**Additional file 1: Table S1.** Sample information.

**Additional file 2: Table S2.** Factor settings.

**Additional file 3: Table S3.** Results of mass uniformity test.

**Additional file 4: Table S4.** Results of response variables from DoE experiment.

**Additional file 5: Table S5.** Post-hoc multiple comparisons.

**Additional file 6: Table S6.** 95% CI for mean (%) release of ART and LUM at different time points.

**Additional file 7: Table S7.** Pair wise comparison of means of AUC, DE and MDT of ART and LUM from different FDC ART/LUM products.

## Data Availability

The datasets used and/or analysed during the current study are available from the corresponding author on request.

## Abbreviations

ART: Artemether
LUM: Lumefantrine
FDC: Fixed dose combination
WHO: World Health Organization
ICH: The International Conference for Harmonisation of Technical Requirements for Pharmaceuticals for Human Use
