# Supplementary material for: Development of a dissolution method for lumefantrine and artemether in immediate release fixed dose artemether/lumefantrine tablets
Source: Malar J. 2020 Apr 7;19:139. doi: 10.1186/s12936-020-03209-5 (PMC7140584; doi:10.1186/s12936-020-03209-5)
Supplement: Supplementary file 1 — Additional file 1: Table S1. Sample information. [file 12936_2020_3209_MOESM1_ESM.docx]

**Table. Sample information for the FDC ART/LUM products**.

| # | Product name | Strength (mg) | Batch no. | Expiry date | Manufactures | Country of origin |
| --- | --- | --- | --- | --- | --- | --- |
| 1 | Artemether-lumefantrine | 20/120 | DYI478058 | 12/2019 | IPCA Laboratories Ltd | India |
| 2 | Artemether-lumefantrine | 20/120 | DYI473315 | 04/2015 | IPCA Laboratories Ltd | india |
| 3 | Comether^®^ | 20/120 | 17EA | 04/2020 | Kunming Pharmaceutical group | China |
| 4 | Artemine^®^ | 20/120 | 24206 | 10/2020 | Addis Pharmaceutical Factory PLC | Ethiopia |
| 5 | Artel-L^®^ | 20/120 | AT8216 | 09/2020 | AKriti Pharmaceutical Pvt Ltd | India |
