# Supplementary material for: Development of a dissolution method for lumefantrine and artemether in immediate release fixed dose artemether/lumefantrine tablets
Source: Malar J. 2020 Apr 7;19:139. doi: 10.1186/s12936-020-03209-5 (PMC7140584; doi:10.1186/s12936-020-03209-5)
Supplement: Supplementary file 2 — Additional file 2: Table S2. Factor settings. [file 12936_2020_3209_MOESM2_ESM.docx]

**Table**. Factors level settings and response variables of 2^3^ full factorial design of experiment.

| Factors | Level | |
| --- | --- | --- |
|  | Low (-1) | High (+1) |
| Agitation speed (A1) | 50 rpm | 100 rpm |
| pH (A2) | 1.3 | 3.3 |
| Surfactant concentration (A3) | 0.5% | 1.5% |
| Response variables | Target | |
| R1: Q at 60 minute^(1)^ | Q ≥ 80% | |
| R2: Range of duplicate values at 60 min^(1)^ | < 5% | |
| R3: Fitness to dissolution model^(2)^ | R^2^ ≥ 0.999 | |
| R4: Mean values as central measure of the four dissolution time points (30, 60, 90 and 120 min) | Q at 30, 60, 90 min ≥60%, 80%, 90% and 120 min (95-100%), respectively | |
| R5: Relative standard deviation (%RSD) of replicates | ≤ 2% | |
| R6: Dissolution Efficiency (DE)^(3)^ | ≥69.60 | |
| R7: Mean dissolution time^(4)^ | ≤32.84 | |

(1) Target or optimum value or range.

(2) Dissolution model applied is Weibull.

(3) DE (%)= $DE=\int_{0}^{t} \frac{y x dt}{y100 x t}x100$ (the percentage ratio of the area under the dissolution curve up to time t to that of the area of the rectangle described by 100% dissolution at the same time point).

(4) Calculated as $MDT=\frac{AUC}{Qmax}$ where AUC is area under dissolution time curve, Qmax is the maximum amount of drug substance dissolved in a given time.

N.B. DE and MDT were calculated based on the assumptions given in R4.
