# Supplementary material for: Development of a dissolution method for lumefantrine and artemether in immediate release fixed dose artemether/lumefantrine tablets
Source: Malar J. 2020 Apr 7;19:139. doi: 10.1186/s12936-020-03209-5 (PMC7140584; doi:10.1186/s12936-020-03209-5)
Supplement: Supplementary file 3 — Additional file 3: Table S3. Results of mass uniformity test. [file 12936_2020_3209_MOESM3_ESM.docx]

**Table .** Results of mass uniformity of 5 commercially available FDC ART/LUM products.

| # | ART/LUM | | | ART/LUM* | | Artemine^®^ | Comether^®^ | | Artel-L^®^ |
| --- | --- | --- | --- | --- | --- | --- | --- | --- | --- |
| 1 | 239.40 | | | 249.5 | | 291.60 | 249.50 | | 289.40 |
| 2 | 243.30 | | | 249.5 | | 290.30 | 246.30 | | 287.60 |
| 3 | 244.40 | | | 248.5 | | 280.00 | 245.80 | | 287.80 |
| 4 | 244.20 | | | 251.4 | | 312.50 | 245.20 | | 285.00 |
| 5 | 240.80 | | | 250.3 | | 299.00 | 251.10 | | 285.30 |
| 6 | 239.60 | | | 249.9 | | 295.80 | 248.20 | | 287.80 |
| 7 | 245.60 | | | 251.9 | | 279.90 | 249.20 | | 284.70 |
| 8 | 240.00 | | | 251.8 | | 288.20 | 244.70 | | 286.30 |
| 9 | 249.50 | | | 247.7 | | 274.20 | 248.40 | | 290.30 |
| 10 | 244.80 | | | 250.3 | | 312.40 | 244.50 | | 290.10 |
| 11 | 238.00 | | | 250.8 | | 293.00 | 247.00 | | 290.50 |
| 12 | 246.70 | | | 249.9 | | 296.20 | 246.80 | | 287.90 |
| 13 | 249.50 | | | 248.1 | | 285.20 | 247.00 | | 286.80 |
| 14 | 244.40 | | | 251.6 | | 300.80 | 249.40 | | 288.70 |
| 15 | 241.00 | | | 250 | | 290.40 | 249.50 | | 284.10 |
| 16 | 244.00 | | | 249.3 | | 293.10 | 249.40 | | 288.60 |
| 17 | 244.70 | | | 248.7 | | 288.60 | 248.50 | | 284.20 |
| 18 | 245.70 | | | 247.4 | | 282.40 | 250.80 | | 286.90 |
| 19 | 248.00 | | | 252.7 | | 293.50 | 245.30 | | 284.60 |
| 20 | 246.30 | | | 251.9 | | 291.80 | 245.40 | | 290.50 |
| Average | | 244.00 | 250.06 | | 291.95 | | 247.60 | 287.36 | |
| SD | | 3.31 | 1.52 | | 9.67 | | 2.06 | 2.19 | |
| %RSD | | 1.36 | 0.61 | | 3.31 | | 0.83 | 0.76 | |
| Max | | 249.50 | 252.70 | | 312.50 | | 251.10 | 290.50 | |
| Min | | 238.00 | 247.40 | | 274.20 | | 244.50 | 284.10 | |

*****Expired product.
