# Supplementary material for: Development of a dissolution method for lumefantrine and artemether in immediate release fixed dose artemether/lumefantrine tablets
Source: Malar J. 2020 Apr 7;19:139. doi: 10.1186/s12936-020-03209-5 (PMC7140584; doi:10.1186/s12936-020-03209-5)
Supplement: Supplementary file 4 — Additional file 4: Table S4. Results of response variables from DoE experiment. [file 12936_2020_3209_MOESM4_ESM.docx]

**Table. Results of response variables from DoE experiment.**

| Experimental design matrix | | | Response variables | | | | | | | | | |
| --- | --- | --- | --- | --- | --- | --- | --- | --- | --- | --- | --- | --- |
| A1 | A2 | A3 | R1: Q (%) at 60 min | R2: Range (%) of duplicate values at 60 min | R3: Fitness to traditional model | R4: Mean values as central measure of the four dissolution time points (30, 60, 90 and 120 min) | | | | R5: Relative standard deviation (%RSD) of replicates (min-max) | R6: DE | R7: Mean dissolution time |
|  |  |  | Target | | | | | | | | | |
|  |  |  | Q≥80% (mean, n=2) | < 5% | Weibull (R^2^≥0.999) | Q=60% at 30 min (mean, n=2) | Q=80% at 60 min (mean, n=2) | Q=90% at 90 min (mean, n=2) | Q=100% at 120 min (mean, n=2) | %RSD≤2 (n=2) | Target (≥69.60) | Target (≤32.84 ) |
| ART API | | | | | | | | | | | | |
| 50 | 1.3 | 0.5 | 46.61 | 0 | 0.998 | 44.73 | 46.61 | 48.77 | 51.87 | 0-3.55 | 41.5 | 23.96 |
| 50 | 3.3 | 0.5 | 54.49 | 1.03 | 0.999 | 35.86 | 54.49 | 61.88 | 70.64 | 1.90-15.69 | 46.88 | 40.35 |
| 50 | 1.3 | 1.5 | 58.31 | 2.84 | 0.999 | 46.93 | 58.31 | 69.05 | 74.7 | 0.08-5.70 | 52.91 | 35 |
| 50 | 3.3 | 1.5 | 51.75 | 6.44 | 0.999 | 45.88 | 51.75 | 68.09 | 85.85 | 2.13-12.45 | 52.15 | 47.09 |
| 100 | 1.3 | 0.5 | 67.71 | 0.72 | 0.998 | 64.58 | 67.71 | 71.68 | 77.96 | 0.22-1.83 | 60.73 | 26.5 |
| 100 | 3.3 | 0.5 | 90.78 | 3.04 | 0.999 | 77.71 | 90.78 | 91.25 | 96.16 | 0-3.35 | 76.95 | 23.96 |
| 100 | 1.3 | 1.5 | 85.12 | 0.59 | 0.998 | 80.88 | 85.12 | 86.62 | 87.82 | 0.62-0.69 | 74.13 | 18.7 |
| 100 | 3.3 | 1.5 | 94.27 | 1.56 | 0.997 | 87.65 | 94.27 | 97.11 | 100.19 | 0.88-1.70 | 82.25 | 21.29 |
| LUM API | | | | | | | | | | | | |
| 50 | 1.3 | 0.5 | 81.08 | 2.38 | 0.999 | 70.3 | 81.08 | 86.11 | 92.83 | 0.18-2.94 | 70.97 | 28.25 |
| 50 | 3.3 | 0.5 | 14.33 | 2.13 | 0.999 | 9.83 | 14.33 | 17.45 | 20.6 | 9.25-16.27 | 12.97 | 44.41 |
| 50 | 1.3 | 1.5 | 37.78 | 0.13 | 0.998 | 33.99 | 37.78 | 39.63 | 40.59 | 0.35-0.85 | 32.92 | 22.65 |
| 50 | 3.3 | 1.5 | 6.4 | 0.37 | 0.999 | 4.14 | 6.4 | 8.25 | 10.56 | 0.03-5.86 | 60.19 | 51.62 |
| 100 | 1.3 | 0.5 | 85.2 | 1.74 | 0.999 | 74.82 | 85.2 | 90.53 | 94.96 | 0.97-5.76 | 74.5 | 25.84 |
| 100 | 3.3 | 0.5 | 4.77 | 0.26 | 0.999 | 3.68 | 4.77 | 7.07 | 7.39 | 0.01-8.68 | 48.05 | 42.02 |
| 100 | 1.3 | 1.5 | 93.28 | 0.03 | 0.999 | 87.24 | 93.28 | 96.68 | 99.84 | 0.04-4.92 | 81.77 | 21.7 |
| 100 | 3.3 | 1.5 | 20.36 | 1.12 | 0.999 | 13.05 | 20.36 | 26.24 | 28.48 | 2.04-5.54 | 18.47 | 42.15 |
