# Supplementary material for: Development of a dissolution method for lumefantrine and artemether in immediate release fixed dose artemether/lumefantrine tablets
Source: Malar J. 2020 Apr 7;19:139. doi: 10.1186/s12936-020-03209-5 (PMC7140584; doi:10.1186/s12936-020-03209-5)
Supplement: Supplementary file 5 — Additional file 5: Table S5. Post-hoc multiple comparisons. [file 12936_2020_3209_MOESM5_ESM.docx]

**Table. Post-hoc test multiple comparisons on release of ART and LUM at 60 min from FDC ART/LUM products.**

| **Products** | | **p-value** | |
| --- | --- | --- | --- |
|  |  | **ART** | **LUM** |
| ARL | ARM | 0.194 | 0.000 |
|  | COM | 0.000 | 0.000 |
|  | IPCA | 0.959 | 0.002 |
|  | IPCAE | 0.078 | 0.126 |
| ARM | ARL | 0.194 | 0.000 |
|  | COM | 0.000 | 0.000 |
|  | IPCA | 0.051 | 0.557 |
|  | IPCAE | 0.098 | 0.019 |
| COM | ARL | 0.000 | 0.000 |
|  | ARM | 0.000 | 0.000 |
|  | IPCA | 0.000 | 0.000 |
|  | IPCAE | 0.000 | 0.000 |
| ART/LUM | ARL | 0.959 | 0.002 |
|  | ARM | 0.05 | 0.557 |
|  | COM | 0.000 | 0.000 |
|  | IPCAE | 0.017 | 0.373 |
| ART/LUM-E | ARL | 0.078 | 0.126 |
|  | ARM | 0.989 | 0.019 |
|  | COM | 0.000 | 0.000 |
|  | IPCA | 0.017 | 0.373 |

ARL: Artel-L^®^, ARM: Artemine^®^, COM: Comether^®^, ART/LUM: Artemether/lumefantrine (unexpired), ART/LUM-E: Artemether/lumefantrine (expired). Tukey’s at 5% significance level was used.
