# Supplementary material for: Development of a dissolution method for lumefantrine and artemether in immediate release fixed dose artemether/lumefantrine tablets
Source: Malar J. 2020 Apr 7;19:139. doi: 10.1186/s12936-020-03209-5 (PMC7140584; doi:10.1186/s12936-020-03209-5)
Supplement: Supplementary file 6 — Additional file 6: Table S6. 95% CI for mean (%) release of ART and LUM at different time points. [file 12936_2020_3209_MOESM6_ESM.docx]

**Table. The results of 95%CI for the mean (%) release of ART in FDC ART/LUM products at each time points**.

| **Time (min)** | **Product** | **Mean (%)** | **SE** | **95% CI for mean** | |
| --- | --- | --- | --- | --- | --- |
|  |  |  |  | **Lower bound** | **Upper bound** |
| 30 | Artel-L^®^ | 72.34 | 0.61 | 70.76 | 73.92 |
|  | Artemine^®^ | 79.61 | 0.56 | 78.16 | 81.06 |
|  | Comether^®^ | 60.66 | 0.52 | 59.33 | 61.99 |
|  | ART/LUM | 66.01 | 0.39 | 65.02 | 67.00 |
|  | ART/LUM-E | 73.10 | 0.49 | 71.83 | 74.37 |
| 60 | Artel-L^®^ | 81.90 | 0.48 | 80.67 | 83.12 |
|  | Artemine^®^ | 83.49 | 0.29 | 82.75 | 84.23 |
|  | Comether^®^ | 63.60 | 0.38 | 62.62 | 64.58 |
|  | ART/LUM | 81.41 | 0.79 | 79.39 | 83.44 |
|  | ART/LUM-E | 83.83 | 0.42 | 82.75 | 84.91 |
| 90 | Artel-L^®^ | 84.82 | 0.48 | 83.60 | 86.05 |
|  | Artemine^®^ | 86.89 | 0.33 | 86.05 | 87.73 |
|  | Comether^®^ | 65.72 | 0.41 | 64.66 | 66.78 |
|  | ART/LUM | 87.03 | 1.20 | 83.94 | 90.11 |
|  | ART/LUM-E | 86.85 | 0.52 | 85.52 | 88.18 |
| 120 | Artel-L^®^ | 87.45 | 0.31 | 86.66 | 88.24 |
|  | Artemine^®^ | 94.66 | 1.06 | 91.94 | 97.39 |
|  | Comether^®^ | 78.85 | 0.35 | 77.96 | 79.74 |
|  | ART/LUM | 98.23 | 0.53 | 96.87 | 99.59 |
|  | ART/LUM-E | 89.41 | 0.38 | 88.42 | 90.39 |

ART/LUM: Artemether/lumefantrine (unexpired), ART/LUM-E: Artemether/lumefantrine (expired).

Table. The results of 95%CI for the mean (%) release of LUM in FDC ART/LUM products at each time points.

| Time (min) | Product | Mean (%) | SE | 95% CI for mean | |
| --- | --- | --- | --- | --- | --- |
|  |  |  |  | Upper bound | Lower bound |
| 30 | Artel-L^®^ | 69.83 | 0.45 | 68.67 | 71.00 |
|  | Artemine^®^ | 76.24 | 0.40 | 75.21 | 77.26 |
|  | Comether^®^ | 46.57 | 1.01 | 43.96 | 49.18 |
|  | ART/LUM | 78.10 | 0.60 | 76.57 | 79.63 |
|  | ART/LUM-E | 66.93 | 0.94 | 64.51 | 69.35 |
| 60 | Artel-L^®^ | 88.82 | 0.98 | 86.29 | 91.34 |
|  | Artemine^®^ | 84.03 | 0.26 | 83.36 | 84.70 |
|  | Comether^®^ | 60.68 | 0.52 | 59.33 | 62.03 |
|  | ART/LUM | 85.27 | 0.59 | 83.75 | 86.80 |
|  | ART/LUM-E | 86.78 | 0.29 | 86.04 | 87.53 |
| 90 | Artel-L^®^ | 96.91 | 0.67 | 95.18 | 98.64 |
|  | Artemine^®^ | 91.66 | 0.55 | 90.25 | 93.06 |
|  | Comether^®^ | 62.50 | 0.87 | 60.26 | 64.73 |
|  | ART/LUM | 90.06 | 0.87 | 87.82 | 92.29 |
|  | ART/LUM-E | 87.76 | 0.29 | 87.00 | 88.52 |
| 120 | Artel-L^®^ | 98.54 | 0.34 | 97.67 | 99.40 |
|  | Artemine^®^ | 95.69 | 0.72 | 93.84 | 97.53 |
|  | Comether^®^ | 66.08 | 0.74 | 64.18 | 67.97 |
|  | ART/LUM | 95.80 | 0.50 | 94.51 | 97.10 |
|  | ART/LUM-E | 90.83 | 0.55 | 89.43 | 92.24 |

ART/LUM: Artemether/lumefantrine (unexpired), ART/LUM-E: Artemether/lumefantrine (expired).
