# Supplementary material for: Development of a dissolution method for lumefantrine and artemether in immediate release fixed dose artemether/lumefantrine tablets
Source: Malar J. 2020 Apr 7;19:139. doi: 10.1186/s12936-020-03209-5 (PMC7140584; doi:10.1186/s12936-020-03209-5)
Supplement: Supplementary file 7 — Additional file 7: Table S7. Pair wise comparison of means of AUC, DE and MDT of ART and LUM from different FDC ART/LUM products. [file 12936_2020_3209_MOESM7_ESM.docx]

**Table.** Results showing pair-wise comparison of the means of AUC, DE and MDT of different FDC ART/LUM products.

| Drugs | AUC |
| --- | --- |
| Artemine^®^ | 5002.2^a^ |
| ART/LUM-E | 4914.2^a^ |
| Artel-L^®^ | 4814.3^b^ |
| ART/LUM | 4738.0^b^ |
| Comether^®^ | 3803.6^c^ |
|  | DE |
| Artemine^®^ | 74.3^a^ |
| ART/LUM-E | 72.1^b^ |
| ART/LUM | 71.1^bc^ |
| Artel-L^®^ | 70.8^c^ |
| Comether^®^ | 57.3^d^ |
|  | MDT |
| ART/LUM | 33.1^a^ |
| Comether^®^ | 32.7^a^ |
| Artemine^®^ | 25.7^b^ |
| Artel-L^®^ | 22.9^c^ |
| ART/LUM-E | 22.9^c^ |

Mean separation by Tukey’s at 5% significance level. Pairs of treatments that are not significantly different from one another share the same letter.
